# CentiServer: A Comprehensive Resource, Web-Based Application and R Package for Centrality Analysis

**DOI:** 10.1371/journal.pone.0143111

**Published:** 2015-11-16

**Authors:** Mahdi Jalili, Ali Salehzadeh-Yazdi, Yazdan Asgari, Seyed Shahriar Arab, Marjan Yaghmaie, Ardeshir Ghavamzadeh, Kamran Alimoghaddam

**Affiliations:** 1 Hematology, Oncology and SCT Research Center, Tehran University of Medical Sciences, Tehran, Iran; 2 School of Biological Sciences, Institute for Research in Fundamental Sciences (IPM), Tehran, Iran; 3 Department of Biophysics, Faculty of Biological Sciences, Tarbiat Modares University, Tehran, Iran; Peking University Health Science Center, CHINA

## Abstract

Various disciplines are trying to solve one of the most noteworthy queries and broadly used concepts in biology, essentiality. Centrality is a primary index and a promising method for identifying essential nodes, particularly in biological networks. The newly created CentiServer is a comprehensive online resource that provides over 110 definitions of different centrality indices, their computational methods, and algorithms in the form of an encyclopedia. In addition, CentiServer allows users to calculate 55 centralities with the help of an interactive web-based application tool and provides a numerical result as a comma separated value (csv) file format or a mapped graphical format as a graph modeling language (GML) file. The standalone version of this application has been developed in the form of an R package. The web-based application (CentiServer) and R package (centiserve) are freely available at http://www.centiserver.org/

## Introduction

Network biology and related approaches are fundamental tools used for simplification, reconstruction, analysis, and comprehension of complex behavior in biological systems. Finding the most relevant elements in a network is crucial in network analysis. In various fields, this concept is discussed under different terms such as influential users, influential spreaders, authorities, hubs, key players, spread nodes, essentiality, lethality, and centrality. Although the centrality concept, which has its origin in social sciences, has been adopted into biology [1], there is no consensus among biologists regarding its definition and applications. Indeed, the difference between biological and other types of networks has prompted extensive research efforts aimed at developing more appropriate and reliable methods for the generalization of the centrality concept that could enable its applications in biology. For instance, systems biologists interpreted the centrality, essentiality, and lethality equivalents based on the centrality–lethality rule [2]. According to such interpretations, essential genes that show correlations with human disease genes are suitable candidates for biomarkers and drug targets. Many efforts performed for improving the existing methods and developing new insights of centrality usage, e.g., using percolation [3], the game theory [4], and perturbation [5] to prepare an effective new centrality index.

There are applications for network analysis on different platforms. Majority of these tools compute classical and common centralities, with a maximum of less than 20 computable measures. We categorized these tools into five groups. Group 1 consisted of the tools that are in the form of a standalone software such as CentiBin, which calculates 16 indices [6]. Group 2 consisted of plug-ins, packages, and toolboxes such as igraph, CentiLib, and CentiScaPe, which calculate 13, 14, and 8 indices, respectively [7–9]. Group 3 consisted of libraries and/or frameworks that can be used via programming languages such as Java and C/C++ (S1 File). The implementation codes proposed by developers such as LeaderRank [10] formed group 4. Algorithms provided by the author(s) such as the motif-based centrality [11] formed group 5. In addition to these tools, some centralities, such as the neighborhood functional centrality, have been coined without any proposed computational approaches [12]. The lack of user-friendly and comprehensive software products for computing some centralities have limited their use. However, comparing the capability and efficiency of all measures is not practical. Gathering available studies as an applicable, especially interactive, website could undoubtedly be a good solution for the mentioned problems. In addition, this not only results in saving researchers’ time but also can be an efficient tool for the creation of new ideas. According our knowledge, there is no complete and comprehensive collection of all current measures in either application that calculates the greater number of measures.

## Methods and Implementation

This comprehensive encyclopedia mainly covers fundamental centrality definitions and computational algorithms in the field of network biology. Nearly all papers related to centrality were carefully reviewed for this survey. To make the materials accessible, a web page of pertinent materials, tools (by which the given measures are calculable), and relevant references was created. At present, the encyclopedia contains 113 entries.

### User Interface and Application

For the computation of centralities, a web-based application was developed employing the R [8] programming language and the igraph packages [13]. While some measures are computable by igraph, others are implemented in the R programming environment. Currently 55 measures and its variants are calculable. Users can upload a network file to the server in different formats and create a job after selecting the given measures and determining its option, if required. The job queue is designed to hold many tasks on the server. A specific ID is assigned to each job, which allows the tracing of the given job by the user and the downloading of the calculated output. The optional registering system allows the users to have access to their uploaded networks and recalculation use without needing to upload them again. The list of jobs, their status, and the results are available under the created jobs section.

Results of the centrality-measured calculation are provided in two formats. The numerical result of the calculated centrality can be downloaded in a csv file format. The computed result will be mapped into a GML graph file format for downloading and online visualization.

Additionally, we have prepared a list of available tools for centrality calculation and have categorized these tools based on their platforms and licenses. The accessing addresses and the measures, which are calculable by them, have been presented (S1 File). The measures page allows the users to submit their comments and share their experiences and ideas (S2 File).

### Centrality List Page

All centrality indices were listed alphabetically with a link to their specific page. Furthermore, indices that are more applicable in biological networks were categorized into separate list. The brief definition of each centrality has been explained according to their related source and all references mentioned.

### Create Job Page

A step-by-step process was designed for creating a new job and calculating network centralities via a web-based application. Each job was queued on server for calculation. In step one, users will upload his/her network file. Supported formats for network file include Edge list (.txt), Pajek (.net), UCINET (.dl), GML (.gml), and GraphML (.graphml). For demo and/or test purposes, two sample networks, Human immunodeficiency virus network from BioGRID and Zachary's karate club network, are selectable. Other network features such as network directionality (undirected or directed), unweighted or weighted option for removal of multiple edges, and loops have been specified in this step. In step two, users will select one or multiple centrality(s) for calculation and specify each index option(s) if applicable. Finally, in step three, users will enter an optional description for the job and will create the job by clicking the “Create job” button. After creation, the job will stay on queue for calculation and web-based tool provides a job unique ID for future follow-up. Users can see job status and result files for download. If users create an optional account, all of uploaded network files, created jobs and its results will be available in users profile section.

### Centrality Software Page

This page lists totally 43 stand-alone applications, libraries, packages, plugins, and toolboxes that calculate centrality measures. This list is categorized alphabetically and according to system platforms. Additionally, a brief description about each tool and access link has been provided. Some other features like platform, license type, and publication reference are also provided.

### Comment Function

A commenting system at the bottom of each centrality page allows researchers to comment on the page, related measures, and share their experiences or make any suggestions.

### R Package

We have also developed centiserve, an R package for the statistical R software [8], which contain 34 functions for calculating centrality indices. The centiserve is igraph package-dependent and it uses igraph as the graph framework. The R software is open source and cross-platform, and is available for free through multiple operating systems. The centiserve package is freely available at http://www.centiserver.org. Despite web applications that have some limitations in network size due to shared resources, use of centiserve R package provides the ability to calculate centrality indices for any size of network and only has a limit based on local system resources.

#### Using Package Centiserve

First, download the package according to an operating system, “Package source” for Linux and “Windows binaries” for windows system via this address, http://www.centiserver.org/?q1=rpackage. Then, use the install.packages() command as below: install.packages(path_to_file, repos = NULL, type = "source") where path_to_file would represent the full path and file name. In the windows operating system and RGui, select Install package(s) from local zip files under the Packages menu. After installation, use the below command to see complete helps for centralities:

> library("centiserve")

> help(package = "centiserve")

### Centrality Measures

The following node centralities are calculable by CentiServer: Alpha centrality, Average distance, Bargaining centrality (Bonacich power), Barycenter centrality, Bottleneck, Bridging centrality, Burt's constraint, Centroid value, Closeness centrality (Freeman and Latora), Closeness vitality, ClusterRank, Communicability betweenness centrality, Community centrality, Cross clique connectivity, Current flow betweenness centrality, Current flow closeness centrality, Dangalchev closeness centrality, Decay centrality, Degree centrality, Diffusion degree, DMNC (Density of Maximum Neighborhood Component), Eccentricity centrality, Eigenvector centrality, Entropy centrality, EPC (Edge Percolated Component), Flow betweenness centrality, Geodesic K-path centrality, Graphlet degree centrality, Hubbell index, Information centrality, Katz status index, K-core decomposition, Kleinberg's (HITS), Laplacian centrality, LeaderRank, Leverage centrality, Lin centrality, Load centrality, Lobby index, Local clustering coefficients, Markov centrality, MNC (Maximum Neighborhood Component), PageRank, Pairwise disconnectivity index, Radiality centrality, SALSA (Stochastic Approach for Link-Structure Analysis), Semi local centrality, Shortest-paths betweenness centrality, Strength (weighted vertex degree), Stress centrality, Subgraph centrality, and Topological coefficient. The centrality indices calculate for undirected or directed and for unweighted or weighted networks.

The other indices which described in site, including ATC (Annotation Transcriptional Centrality), Bayesian centrality, Bipartivity, CCIS, Co-Betweenness centrality, Communicability centrality, Composite centrality, Control centrality, Covertness centrality, Degree sphere centrality, DelayFlow centrality, DFC (Disease Flow Centrality), DSS (Double Screening Scheme), Edge disjoint K-path centrality, Edge weighted, Effectiveness, EGC, Eigentrust, Flux centrality, Fuzzy closeness centrality, Game centrality, Group betweenness centrality, Harary graph centrality, Harmonic centrality, Integration centrality, κ-betweenness centrality, κ-path centrality, KatzR centrality, Kleinberg's centrality, Knotty centrality, KSC (K-Shell and Community Centrality), LA (Local Assortativity), LAC (Local Average Connectivity-Based Method), Maximum influence degree, MCC (Maximal Clique Centrality), Motif-Based centrality, Modularity centrality, Modularity density centrality, NC (Network Centrality), NC (Normalized α-Centrality), Neighbor based centrality, Neighborhood connectivity, Neighborhood coreness centrality, Network motif centrality, NFC (Neighborhood Functional Centrality), PeC centrality, Percolation centrality, Personalized PageRank, Perturbation centrality, Principal component centrality, Random-walk betweenness centrality, Random-walk closeness centrality, Ranking-betweenness centrality, SoECC (Sum of Edge Clustering Coefficient), Straightness centrality, Trust transitivity, TwitterRank, WDC (Weighted Degree Centrality), and Weighted LeaderRank.

### Maintenance and Future Growth

The network centrality in social and biology science is still an open field and we strive to improve currently available measures and also develop new ones. The authors and contributors of CentiServer monitor publish literatures and other scientific sources frequently and will update the site with newly developed indices and methods. The CentiServer site is hosted on an academic internet platform that ensures greater accuracy and reliable access to site.

## Applications

Recently, many approaches and models have been developed to computationally identify the essential genes according to two main ideas. 1) Essential genes have a common feature in all organisms and they are evolutionary conserved [14]. This feature is based on functional importance, gene duplicability, and mRNA expression level [15]. 2) The network approach (graph theory) can promote an innovative way of thinking in biology [16, 17]. According to this method, after reconstruction of biological networks by high-throughput data, different network analysis methods have been employed to investigate the behavior of such complex large networks. Topological analysis of biological networks using mathematical approaches computes centrality indices and finds essential nodes. The concept of essential genes (or their products; essential proteins) has been widely used in various fields of biological sciences. These genes are vital to maintain cellular life and their deletion will result in lethality or infertility [18].

Centrality indices are a local network property that ranks the graph nodes. The higher the rank, the more important the node is in the network and may play key roles in controlling cellular responses [19].

**Fig 1:**
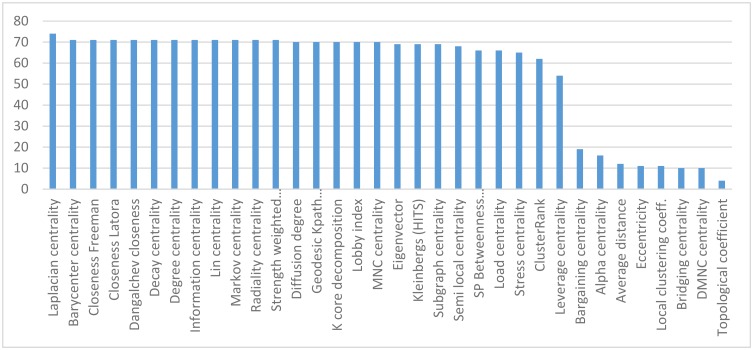
The percentage of essential proteins in the top 100 ranked proteins by various centrality indices.

As a simple illustration, we downloaded the protein-protein interaction network of *Escherichia coli K12* from STRING (Search Tool for the Retrieval of Interacting Genes/Proteins) [20] version 9.1. Only interactions with an experimental score were selected. The loops and parallel edges were removed and the maximum connected component that contains 3138 vertexes (proteins) and 20309 edges (interactions) was selected. The essential proteins of *E*. *coli* were obtained from the Online GEne Essentiality (OGEE) database [21]. Thirty four centrality indices were calculated for given the network by CentiServe. The percentage of essential proteins in the top 100 ranked proteins by various centrality indices are displayed in Fig 1. The best indices were Laplacian centrality, Barycenter centrality, and 3 variations of closeness centrality, which determined about 71–74 essential proteins among top 100 ranked proteins. Previously Schoch and Brandes showed that closeness centrality is an appropriate measure for predicting essential nodes in biological networks [22]. In our study, in addition to confirming previous results, we concluded that the variants of closeness centrality index led to the same results.

Each centrality measure provides an ordered list of proteins (S3 File). For combining these ordered lists into an overall top 20 proteins list, which most likely would be more essential than any individual list by itself, we implemented the rank aggregation method in the RankAggreg R package [23]. For this purpose we applied the RankAggreg function by a Genetic Algorithm and the Spearman distance parameters to 34 rows of ordered lists. After adequate iterations, the optimal ordered list that converged all ordered lists with a minimum objective function score of 53.08 was: *groL* (b4143), *dnaK* (b0014), *rplB* (b3317), *rpsD* (b3296), *rpsB* (b0169), *rplD* (b3319), *rplA* (b3984), *rpsE* (b3303), *rpsC* (b3314), *htpG* (b0473), *tufB* (b3980), *tufA* (b3339), *fusA* (b3340), *rpsG* (b3341), *rpsI* (b3230), *rplC* (b3320), *rplM* (b3231), *rplQ* (b3294), *rpoC* (b3988) and *ybcV* (b0558). Except *rplA* (b3984) and *htpG* (b0473), all of these genes were marked as essential ones previously.

The result of this analysis emphasized that the centrality is an anticipative topological measurement for predicting essential nodes in biological networks, because although about 20 percent of proteins in the protein interaction network of *E*. *coli* were marked as essential genes, the centrality index predicted about 74% of these proteins ranked in the top 100. Moreover, the aggregation method is a promising way for predicting more reliable and accurate essential proteins by unifying all ordered protein lists extracted from each centrality index. Discussing the biological significance of the RankAggreg result is beyond the scope of this article.

## Conclusions

Centrality and essentiality are important and ongoing topics in the areas of social and biological networks. The centrality concept and related algorithms have found wide spread use in identifying essential nodes in a distinct network. With respect to the expansion of networks in all scientific fields, much efforts have been done in this regard. Since these methods have not met the needs, continuing efforts for optimizing indices and creating more efficient indices are expected. Determining the prominent nodes in a network completely depends on our importance definition and the measure we use for a specific purpose. Therefore, selecting an appropriate measure is critical. Additionally, uninformed use of measures without considering the essence of the network and the desired goal could result in misleading results, especially in biology [22]. A query in the resources suggests that the measures of centrality are not limited to the well-known ones. Although several new measures have recently been developed, the majority of them remained unknown. A number of these measures were defined initially based on biology notions, whereas others are optimizations of previous theories. Our goal while developing this encyclopedia and web application tool was to first provide all available centrality values, definitions, calculating methods, algorithms, and related software products in one place. This encyclopedia of centrality with its computational facilities was created to help researchers to make better choices of measures by reviewing the previous works, come up with new ideas, and avoid repetition. The second goal was to develop an application, which calculates more centrality measures in comparison to other tools. Access to all measures will not only help scientists to develop and optimize indices, but also provide an opportunity to calculate more centrality values for meaningful analysis of biological networks.

The site's materials will be kept updated and new contents will be added. The number of computable measures will be increased to improve the capability of the website. Adding practical applications of each index could significantly contribute the functionality of the site.

## Supporting Information

S1 File The list of software products which compute centrality indices and its features. (DOC)

S2 File Screenshots of CentiServer website. (DOC)

S3 File The ordered lists of proteins ranked by each centrality measures. (CSV)
