# Supplementary material for: CentiServer: A Comprehensive Resource, Web-Based Application and R Package for Centrality Analysis
Source: PLoS One. 2015 Nov 16;10(11):e0143111. doi: 10.1371/journal.pone.0143111 (PMC4646361; doi:10.1371/journal.pone.0143111)
Supplement: S1 File — (DOC) [file pone.0143111.s001.doc]

**Supplementary File S1**

**The list of software products which compute centrality indices and its features.**

| **Product** | **Centralities** | **Count** | **Platform** | **License** | **Reference** |
| --- | --- | --- | --- | --- | --- |
| **AllegroGraph**  <http://franz.com/agraph/allegrograph/> AllegroGraph® is a modern, high-performance, persistent graph database. AllegroGraph uses efficient memory utilization in combination with disk-based storage, enabling it to scale to billions of quads while maintaining superior performance. AllegroGraph supports SPARQL, RDFS++, and Prolog reasoning from numerous client applications. | Betweenness centrality Closeness centrality Degree centrality | 3 | Graph database (Linux) | Free  Commercial |  |
| **The Brain Connectivity Toolbox (BCT)**  <http://www.brain-connectivity-toolbox.net/> The BCT contains a large selection of complex network measures in Matlab. These measures are increasingly used to characterize structural and functional brain connectivity datasets. | Edge betweenness centrality K-coreness centrality Node betweenness centrality Node-wise flow coefficients PageRank centrality Participation coefficient Shannon-entropy based diversity coefficient Shortcuts (erange) Spectral measure of centrality Subgraph centrality Within-module degree z-score | 11 | MatLab toolbox | Free  Open source |  |
| **CentiBiN**  <http://centibin.ipk-gatersleben.de/>  CentiBiN is an application for the calculation and visualization of centralities for biological networks. | Bargaining centrality Centroid value Closeness centrality Closeness Vitality Current-Flow Betweenness centrality Current-Flow Closeness centrality Degree centrality Eccentricity centrality Eigenvector centrality HITS Hubbell Index Katz Status Index PageRank Radiality centrality Shortest-Paths Betweenness centrality Stress centrality | 16 | Java standalone | Free |  |
| **CentiLib**  <http://centilib.ipk-gatersleben.de/> CentiLib is a Java-library for the computation and investigation of weighted and unweighted centralities in biological networks. | Betweenness centrality Centroid centrality Closeness centrality Current-flow closeness centrality Current-flow betweenness centrality Degree centrality Eccentricity centrality Eigenvector centrality HITS Hubbel index Katz status index centrality PageRank Radiality centrality Stress centrality | 14 | Java-library / Vanted/Cytoscape | Free  Open source |  |
| **CentiScaPe**  <http://www.cbmc.it/~scardonig/centiscape/centiscape.php> Find the most important nodes in a network, calculating centrality parameters for each node. | Betweenness centrality (vertex/edge) Bridging centrality Centroid centrality Closeness centrality Eccentricity centrality Eigenvector centrality Radiality centrality Stress centrality | 8 | Cytoscape Plugin | Free |  |
| **cyto-Hubba**  <http://hub.iis.sinica.edu.tw/cytohubba/> cytoHubba is a Java plugin for Cytoscape, a facilitated platform for the analysis and visualization of molecular interaction networks based on web application, Hubba. | Betweenness centrality  Bottleneck (BN)  Closeness centrality  Clustering coefficient Degree Density of Maximum Neighborhood Component (DMNC) Double Screening Scheme (DSS) of MNC || DMNC  EcCentricity Edge Percolated Component (EPC) Maximum Neighborhood Component (MNC)  Radiality centrality  Stress centrality | 12 | Cytoscape plugin | Free |  |
| **CytoNCA**  <http://apps.cytoscape.org/apps/cytonca> Providing calculation, evaluation and visualization analysis for several centralities of weighted and unweighted network. | Betweenness centrality Closeness centrality Degree centrality Eigenvector centrality Information centrality Local Average Connectivity-based method (LAC) Network centrality (NC) Subgraph centrality | 8 | Cytoscape Plugin | Free |  |
| **EgoNet**  <http://escoladeredes.net/profiles/blogs/egonet-1> <http://sourceforge.net/projects/egonet/> EgoNet (Egocentric Network Study Software) for the collection and analysis of egocentric social network data.[1] It helps the user to collect and analyse all the egocentric network data (all social network data of a website on the Internet), and provide general global network measures and data matrixes that can be used for further analysis by other software. | Betweenness centrality Closeness centrality Degree centrality | 3 | Java standalone | Free |  |
| **Functional Genomics Assistant (FUGA)**  <http://code.google.com/p/fuga> is a MATLAB toolbox for inference and analysis of biological and cellular networks. | Clustering coefficient (parallelized) Eccentricity centrality Local efficiency Node betweenness centrality (parallelized) Node degree centrality Node eigenvector centrality | 6 | MatLab toolbox | Free  Open source |  |
| **Gephi**  <https://gephi.github.io/> | See product documentation |  | Java standalone | Free  Open Source |  |
| **GraphChi**  <http://graphlab.org/projects/graphchi.html> <https://github.com/GraphChi> Disk-based large-scale graph computation. | Personalized PageRank PageRank | 2 | C++ / Java framework | Free  Open Source |  |
| **GraphStream**  <http://graphstream-project.org/> GraphStream is a Java library for the modeling and analysis of dynamic graphs. You can generate, import, export, measure, layout and visualize them. | Betweenness centrality Centroid Closeness centrality Degree centrality Eccentricity centrality Eigenvector centrality PageRank | 7 | Java framework | Free  Open Source |  |
| **graph-tool**  <http://graph-tool.skewed.de/> Graph-tool is an efficient Python module for manipulation and statistical analysis of graphs (a.k.a. networks). The core data structures and algorithms are implemented in C++. | Authority and hub centralities Betweenness centrality Closeness centrality Eigentrust centrality Eigenvector centrality Katz centrality PageRank Trust transitivity | 8 | Python | Free  Open source |  |
| **GTNA**  <https://www.p2p.tu-darmstadt.de/research/gtna/> GTNA is a Java-based framework that allows for the graph-theoretic analysis of arbitrary network topologies. | Betweenness centrality Local Clustering Coefficient PageRank | 3 | Java framework | Free  Open source |  |
| **igraph**  <http://igraph.org/>  The network analysis package. igraph is a collection of network analysis tools with the emphasis on efficiency, portability and ease of use. | Alpha centrality Average distance Betweenness  centrality Bonacich Power centrality Closeness centrality Constraint centrality Degree centrality Eccentricity centrality Eigenvector centrality Kleinberg centrality PageRank Strength centrality Subgraph centrality | 13 | R package / Python / C Library | Free  Open Source |  |
| **InFlow**  <http://www.orgnet.com/inflow3.html> Software for Social Network Analysis & Organizational Network Analysis. | See product documentation |  | Windows | Commercial |  |
| **Interference**  <http://www.cbmc.it/~scardonig/interference/Interference.php> A tool for virtual experimental network topological analysis. | Betweenness centrality Centroid centrality Closeness centrality Eccentricity centrality Radiality centrality Stress centrality | 6 | Cytoscape Plugin | Free |  |
| **JGraphT-sna**  <https://bitbucket.org/sorend/jgrapht-sna> JGraphT, Social Network Analysis addon. | Brandes betweenness centrality Dangalchev closeness centrality Degree centrality Eigenvector centrality Freeman closeness centrality Fuzzy closeness centrality Geodesic KPath centrality Influence independence centrality Information centrality KPath centrality Latora closeness centrality OrtizArroyo Entropy centrality Random walk betweenness centrality Weighted closeness centrality Weighted degree centrality | 15 | Java framework | Free  Open Source |  |
| **JUNG**  <http://jung.sourceforge.net/> The Java Universal Network/Graph Framework--is a software library that provides a common and extendible language for the modeling, analysis, and visualization of data that can be represented as a graph or network. | Average distance Betweenness centrality Barycenter centrality Closeness centrality Degree centrality Eccentricity centrality Eigenvector centrality HITS HITS with priors Markov centrality PageRank PageRank with priors Random walk betweenness | 13 | Java framework | Free  Open Source |  |
| **ModuLand**  <http://www.linkgroup.hu/modules.php> <http://apps.cytoscape.org/apps/moduland> Modularization method family offering modular hierarchies and adjustable overlaps. | Community centrality Bridgeness Overlap | 2 | Cytoscape Plugin | Free |  |
| **MultiNet**  <http://www.sfu.ca/personal/archives/richards/Multinet/Pages/multinet.htm> MultiNet is a data analysis package that can be used for ordinary data (in which you have a file that has one line of data for each case) and for network data (in which there are two files -- the "node" file describes the individuals and the "link" file describes the connections between individuals). | Betweenness centrality Closeness centrality Degree distributions Eigenvector centrality Influence (Out) centrality Influence (In) centrality Integration centrality Radiality centrality | 8 | Windows | Shareware |  |
| **neo4j**  [https://github.com/neo4j/neo4j/.../centrality](https://github.com/neo4j/neo4j/tree/master/community/graph-algo/src/main/java/org/neo4j/graphalgo/impl/centrality) <http://neo4j.com/> Neo4j is the world's leading graph database. People everywhere are using Neo4j to find graphs in every industry, connecting data to make sense of everything. | Betweenness centrality Closeness centrality Eccentricity centrality Eigenvector centrality Eigenvector centrality arnoldi Eigenvector centrality power Shortest path based centrality Stress centrality | 8 | Windows | Commercial |  |
| **NetMiner**  <http://www.netminer.com/> NetMiner is an premium software tool for Exploratory Analysis and Visualization of Network Data. | See product documentation |  | Windows | Commercial |  |
| **NetVis Module**  <http://www.netvis.org/> The NetVis Module is a free open source web-based tool to analyze and visualize social networks using data from csv files, online surveys, and dispersed teams. | Betweenness centrality Closeness centrality Constraint Degree centrality Efficiency Hierarchy Reachability | 7 | Web based | Free  Open source |  |
| **NetworkAnalyzer**  <http://med.bioinf.mpi-inf.mpg.de/networkanalyzer/> NetworkAnalyzer is a Java plugin for Cytoscape, a software platform for the analysis and visualization of molecular interaction networks. | Betweenness centrality Closeness centrality Clustering coefficient Degree distributions Eccentricity Neighborhood connectivity Radiality Shared neighbors Shortest paths Stress centrality Topological coefficients | 11 | Cytoscape plugin | Free  Open source |  |
| **NetworKit**  <https://networkit.iti.kit.edu/> NetworKit is a growing open-source toolkit for high-performance network analysis. Its aim is to provide tools for the analysis of large networks in the size range from thousands to billions of edges. | ApproxBetweenness centrality Betweenness centrality Degree centrality Eigenvector centrality PageRank Spectral centrality | 5 | Python | Free |  |
| **NetworkX**  <https://networkx.github.io/>  NetworkX is a Python language software package for the creation, manipulation, and study of the structure, dynamics, and functions of complex networks. | Betweenness centrality Closeness centrality Communicability Current-Flow Betweenness centrality Current Flow Closeness centrality Degree centrality Dispersion Eigenvector centrality Load centrality | 9 | Python | Free |  |
| **NodeXL**  <http://nodexl.codeplex.com/> NodeXL: Network Overview, Discovery and Exploration for Excel. | Betweenness centrality Closeness centrality Clustering coefficient Degree distributions Eigenvector centrality PageRank | 6 | Windows | Free  Open source |  |
| **qgraph**  <http://sachaepskamp.com/qgraph> <http://cran.r-project.org/web/packages/qgraph/> qgraph is a package that can be used to plot several types of graphs. It is mainly aimed at visualizing relationships in (psychometric) data as networks to create a clear picture of what the data actually looks like. | Betweenness centrality Closeness centrality Degree centrality | 3 | R package | Free  Open source |  |
| **Pajek**  <http://pajek.imfm.si/> Pajek - Program for Large Network Analysis. | Betweenness centrality Burt’s measure of constraint (structural holes) Closeness centrality Clustering coefficient Degree distributions Hubs-Authorities Summing up Values of Line | 7 | Windows | Free for noncommercial use |  |
| **RBGL**  <http://www.bioconductor.org/packages/release/bioc/html/RBGL.html> A fairly extensive and comprehensive interface to the graph algorithms contained in the BOOST library. | Betweenness centrality kCores | 2 | R package | Free  Open source |  |
| **RINalyzer**  [http://rinalyzer.de/](http://rinalyzer.de/docu/cent_analysis.php) RINalyzer provides a number of important methods for analyzing and visualizing residue interaction networks (RINs). | Current flow betweenness centrality Current flow closeness centrality Random walk betweenness centrality Random walk closeness centrality Shortest path betweenness centrality Shortest path closeness centrality Weighted degree centrality | 7 | Cytoscape Plugin | Free |  |
| **SANTA**  <http://bioconductor.org/packages/release/bioc/html/SANTA.html> Spatial Analysis of Network Associations. This package provides methods for measuring the strength of association between a network and a phenotype. It does this by measuring clustering of the phenotype across the network. Vertices can also be individually ranked by their strength of association with high-weight vertices. | Knode Markov centrality | 2 | R package | Free  Open source |  |
| **SBEToolbox**  <https://github.com/biocoder/SBEToolbox/releases> A Matlab Toolbox for Biological Network Analysis. | Betweenness centrality Bridging centrality Closeness centrality Clustering Coefficient Degree centrality Eccentricity centrality Knotty centrality | 7 | MatLab toolbox | Free  Open source |  |
| **Sentinel Visualizer**  <http://www.fmsasg.com/SocialNetworkAnalysis/>  Sentinel Visualizer integrates Social Network Analysis (SNA) directly into your link chart diagrams so you can quickly generate SNA metrics on your data. | Betweenness centrality Closeness centrality Degree centrality Eigenvector centrality Hub and Authority | 5 | Windows | Commercial |  |
| **sna**  <http://CRAN.R-project.org/package=sna>  A range of tools for social network analysis, including node and graph-level indices, structural distance and covariance methods, structural equivalence detection, network regression, random graph generation, and 2D/3D network visualization. | Betweenness centrality Bonacich power centrality Closeness centrality Degree centrality Eigenvector centrality Flow betweenness centrality Harary graph centrality Information centrality K-core decomposition Load centrality Stress centrality | 11 | R package | Free  Open Source |  |
| **SocNetV**  <http://socnetv.sourceforge.net/> Social Networks Visualizer (SocNetV) is a cross-platform, user-friendly tool for the analysis and visualization of Social Networks. | Betweenness centrality Closeness centrality Degree centrality Degree Prestige (inDegree) Eccentricity centrality Influence Range Closeness centrality Information centrality PageRank Prestige Power centrality Proximity Prestige Stress centrality | 11 | Windows, Linux, Mac | Free  Open Source |  |
| **tnet**  <http://cran.r-project.org/web/packages/tnet/> R package for analyzing weighted, two-mode, and longitudinal networks. | Betweenness centrality (weighted network) Closeness centrality (weighted network) Degree centrality (two-mode network) Degree centrality (weighted network) Distance (two-mode network) Distance (weighted network) | 4 | R package | Free  Open Source |  |
| **UCINET**  <https://sites.google.com/site/ucinetsoftware/> UCINET 6 for Windows is a software package for the analysis of social network data. | Betweenness centrality Bonacich power centrality Closeness centrality Degree centrality Distance weighted fragmentation Eigenvector centrality Flow betweenness centrality Fragmentation centrality Hubs and authorities centrality K-step reach centrality Information centrality Political independence index (pii) Proximal betweenness centrality Reverse closeness centrality | 14 | Windows | Commercial |  |
| **Visone**  <http://visone.info/> Visone is a long-term research project (team), in which models and algorithms to integrate and advance the analysis and visualization of social networks are being developed. | Betweenness centrality Closeness centrality Current-flow centralities Degree centrality Eccentricity centrality Eigenvector centrality Hubs and authorities Pagerank Radiality centrality Status Stress centrality | 7 | Java standalone | Free for academic and research |  |
| **WebGraph**  <http://webgraph.di.unimi.it/> WebGraph is a framework for graph compression aimed at studying web graphs. | Betweenness centrality Closeness centrality Lin's centrality Harmonic centrality Reachable | 5 | Java framework | Free |  |
| **Wolfram**  <http://www.wolfram.com/mathematica/> <http://www.wolframalpha.com/> <http://reference.wolfram.com/language/guide/GraphMeasures.html> | Betweenness centrality Degree centrality Closeness centrality Eccentricity centrality Edge Betweenness centrality Edge Connectivity Eigenvector centrality HITS centrality Katz centrality Local clustering coefficient PageRank centrality Radiality centrality Status centrality SALSA Vertex Connectivity | 14 | Windows | Commercial |  |

**References**

AJ, C. and F, M. (2014) SANTA: Quantifying the Functional Content of Molecular Networks, PLOS Computational Biology, 10, e1003808.

Assenov, Y., et al. (2008) Computing topological parameters of biological networks, Bioinformatics, 24, 282-284.

Bastian, M., Heymann, S. and Jacomy, M. (2009) Gephi: an open source software for exploring and manipulating networks, ICWSM, 8, 361-362.

Batagelj, V. and Mrvar, A. (2002) Pajek—analysis and visualization of large networks. Graph Drawing. Springer, pp. 477-478.

Boldi, P. and Vigna, S. (2004) The webgraph framework I: compression techniques. Proceedings of the 13th international conference on World Wide Web. ACM, New York, NY, USA, pp. 595-602.

Borgatti, S., Everett, M. and Freeman, L. (2002) UCINET 6 for Windows: Software for social network analysis (Version 6.102), Harvard, MA: Analytic Technologies.

Brandes, U.W. (2003) D.(2003).“Visone. Analysis and Visualization of Social Networks”, Special Issue on Graph Drawing Software, Springer-Verlag, Springer Series in Mathematics and Visualization, Springer-Verlag, págs, 321-340.

Butts, C.T. (2014) sna: Tools for social network analysis, R package version, 2.

Carey, V., Long, L. and Gentleman, R. (2010) RBGL: An interface to the BOOST graph library, R package version, 1, 124.

Chen, S.-H., et al. (2009) cyto-Hubba: A Cytoscape plug-in for hub object analysis in network biology. 20th International Conference on Genome Informatics.

Csardi, G. and Nepusz, T. (2006) The igraph software package for complex network research, InterJournal, Complex Systems, 1695.

Doncheva, N.T., et al. (2011) Analyzing and visualizing residue networks of protein structures, Trends in Biochemical Sciences, 36, 179-182.

Drozdov, I., et al. (2011) Functional Genomics Assistant (FUGA): a toolbox for the analysis of complex biological networks, BMC research notes, 4, 462.

Dutot, A., et al. (2007) Graphstream: A tool for bridging the gap between complex systems and dynamic graphs. Emergent Properties in Natural and Artificial Complex Systems. Satellite Conference within the 4th European Conference on Complex Systems (ECCS'2007).

Epskamp, S., et al. (2012) Qgraph: Network visualizations of relationships in psychometric data, Journal of Statistical Software, 48, 1-18.

Gräßler, J., Koschützki, D. and Schreiber, F. (2012) CentiLib: comprehensive analysis and exploration of network centralities, Bioinformatics, 28, 1178-1179.

Hagberg, A., Swart, P. and S Chult, D. (2008) Exploring network structure, dynamics, and function using NetworkX. Los Alamos National Laboratory (LANL).

Junker, B., Koschutzki, D. and Schreiber, F. (2006) Exploration of biological network centralities with CentiBiN, BMC Bioinformatics, 7, 219.

Konganti, K., et al. (2013) SBEToolbox: A Matlab Toolbox for Biological Network Analysis, Evolutionary Bioinformatics, 9, 355-362.

Kyrola, A., Blelloch, G.E. and Guestrin, C. (2012) GraphChi: Large-Scale Graph Computation on Just a PC. OSDI. pp. 31-46.

Naveh, B. (2011) JGraphT a free Java graph library.

O’Madadhain, J., et al. (2005) Analysis and visualization of network data using JUNG, Journal of Statistical Software, 10, 1-35.

Opsahl, T. (2014) tnet Weighted Networks» Node Centrality, R package.

Rubinov, M. and Sporns, O. (2010) Complex network measures of brain connectivity: Uses and interpretations, NeuroImage, 52, 1059-1069.

Scardoni, G., Petterlini, M. and Laudanna, C. (2009) Analyzing biological network parameters with CentiScaPe, Bioinformatics, 25, 2857-2859.

Schiller, B., et al. (2010) GTNA: a framework for the graph-theoretic network analysis. Proceedings of the 2010 Spring Simulation Multiconference. Society for Computer Simulation International, Orlando, Florida, pp. 1-8.

Smith, M., et al. (2010) NodeXL: a free and open network overview, discovery and exploration add-in for Excel 2007/2010, Social Media Research Foundation.

Staudt, C.L., Sazonovs, A. and Meyerhenke, H. (2014) NetworKit: An Interactive Tool Suite for High-Performance Network Analysis, arXiv preprint arXiv:1403.3005.

Szalay-Bekő, M., et al. (2012) ModuLand plug-in for Cytoscape: determination of hierarchical layers of overlapping network modules and community centrality, Bioinformatics, 28, 2202-2204.

Tang, Y., et al. (2014) CytoNCA: A cytoscape plugin for centrality analysis and evaluation of protein interaction networks, Biosystems.

Wolfram Research, I. (2014) Mathematica, Version 10.0, Champaign, IL.
