# Supplementary figures and images for: CentiServer: A Comprehensive Resource, Web-Based Application and R Package for Centrality Analysis
Source: PLoS One. 2015 Nov 16;10(11):e0143111. doi: 10.1371/journal.pone.0143111 (PMC4646361; doi:10.1371/journal.pone.0143111)

**Supplementary File S2**

Screenshots of CentiServer website:


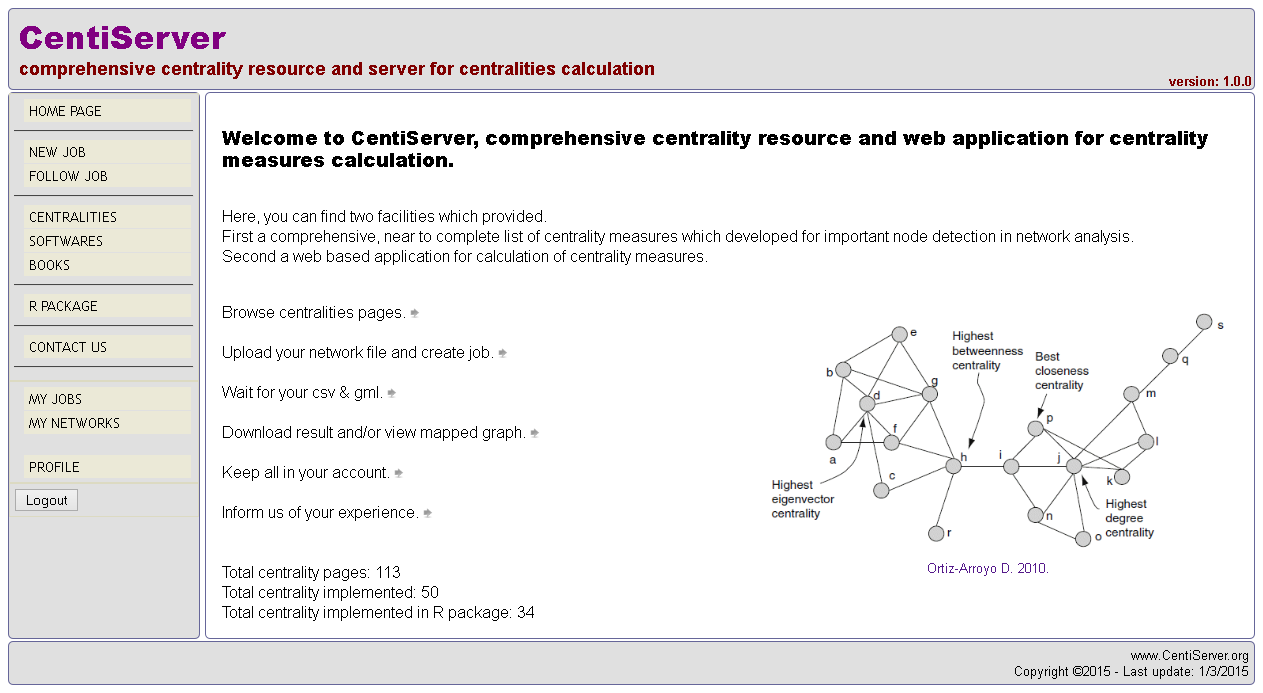


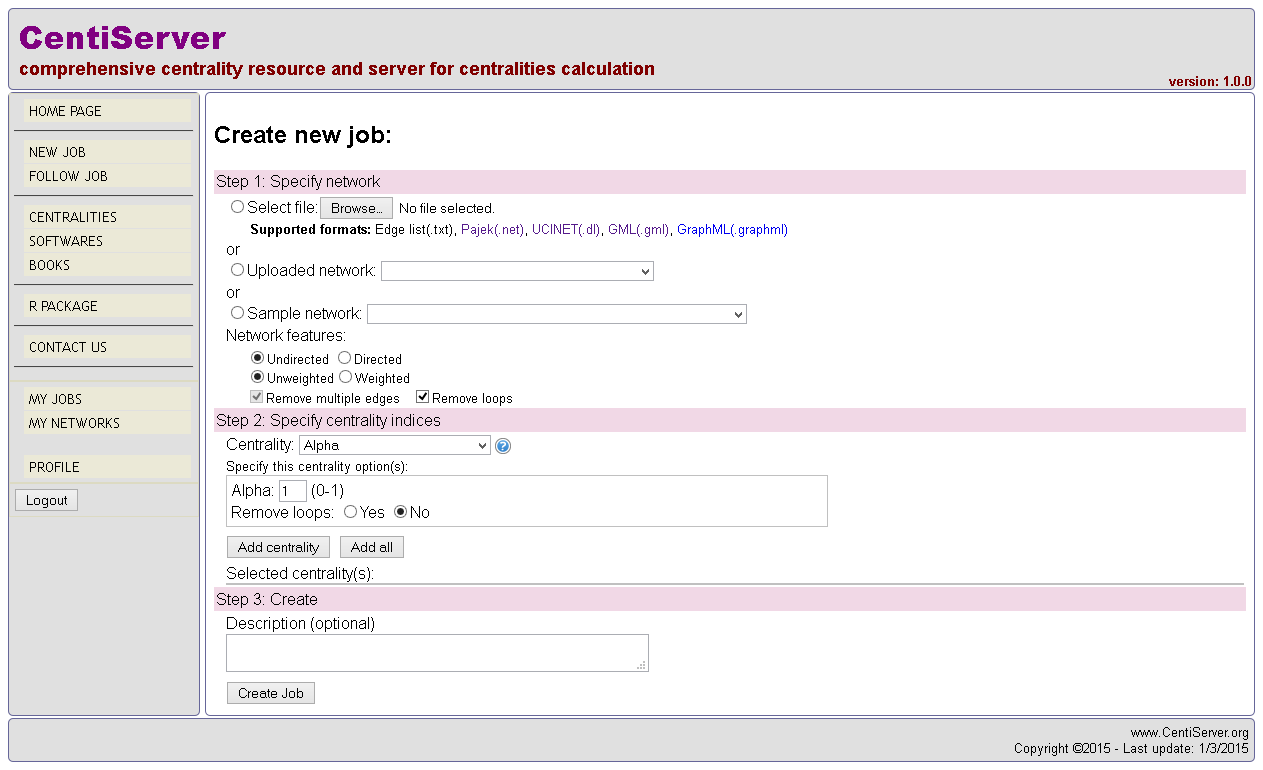


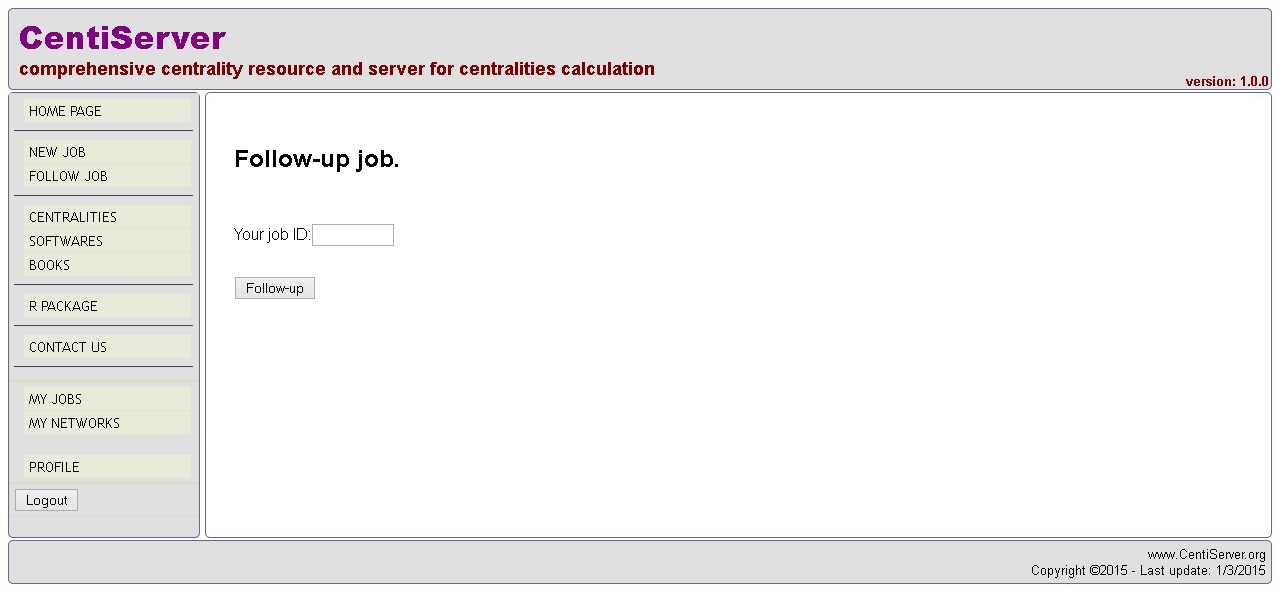


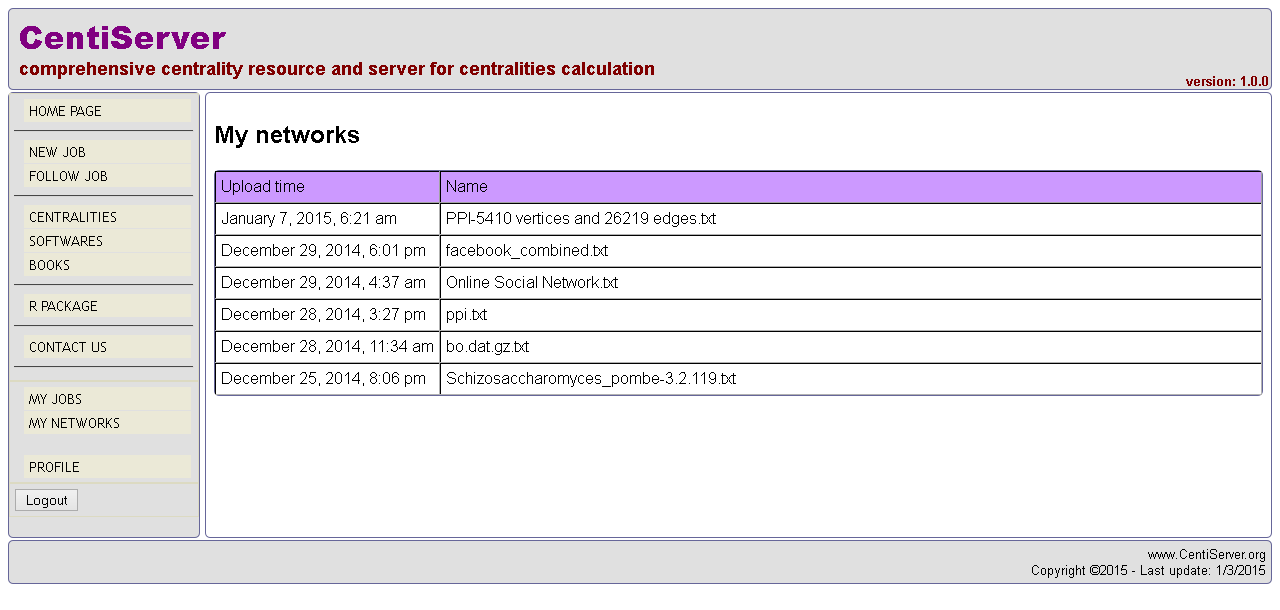


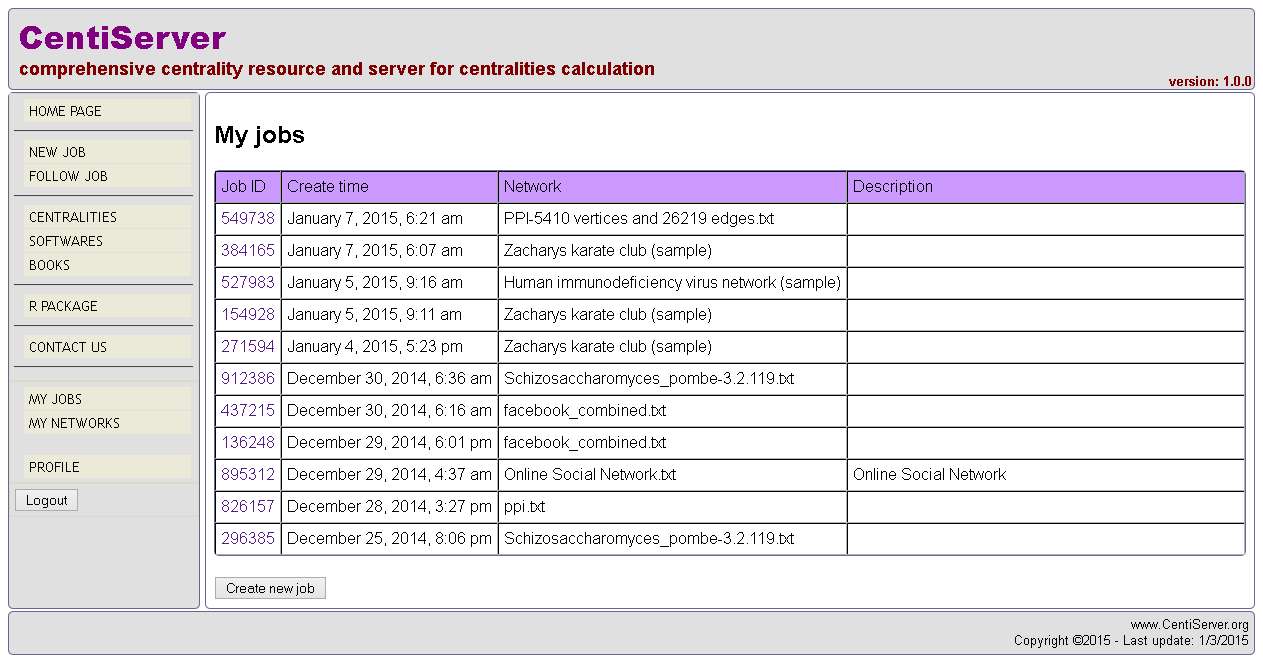


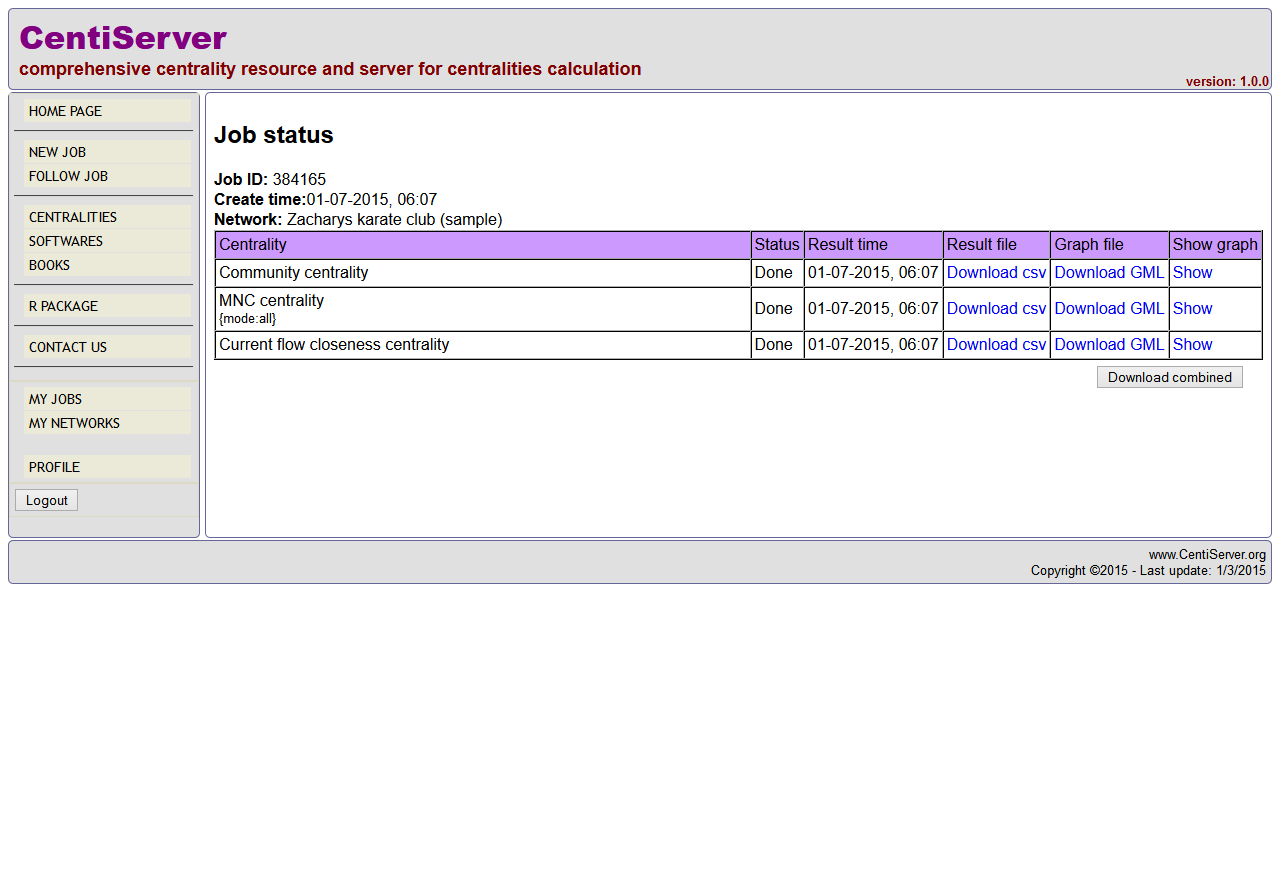


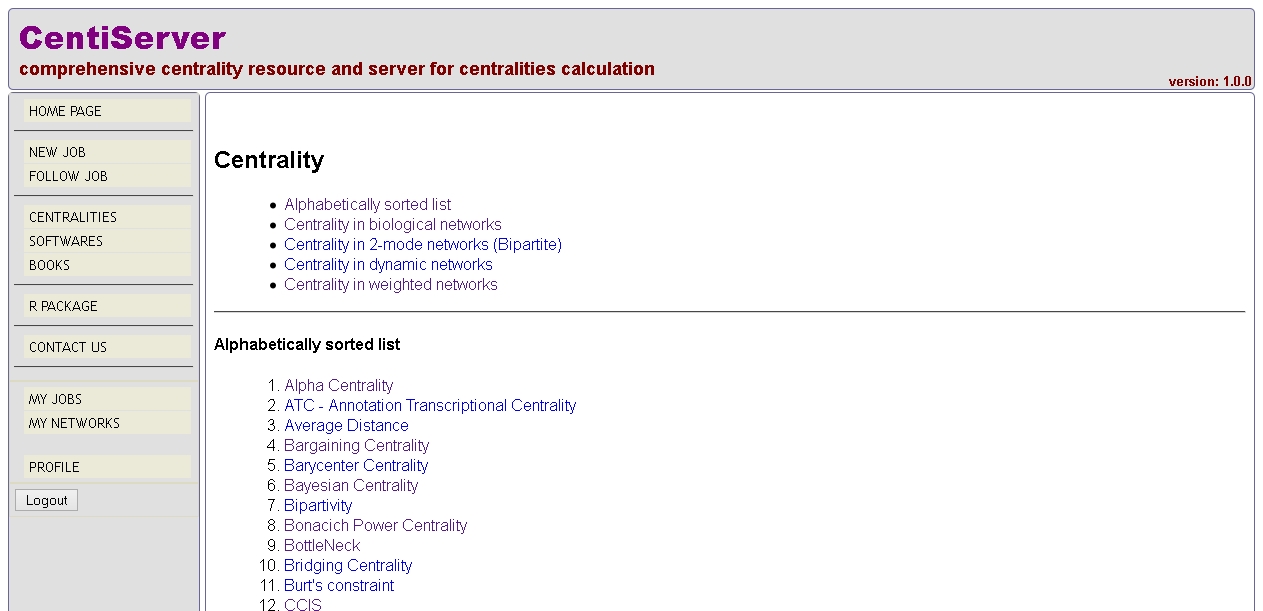


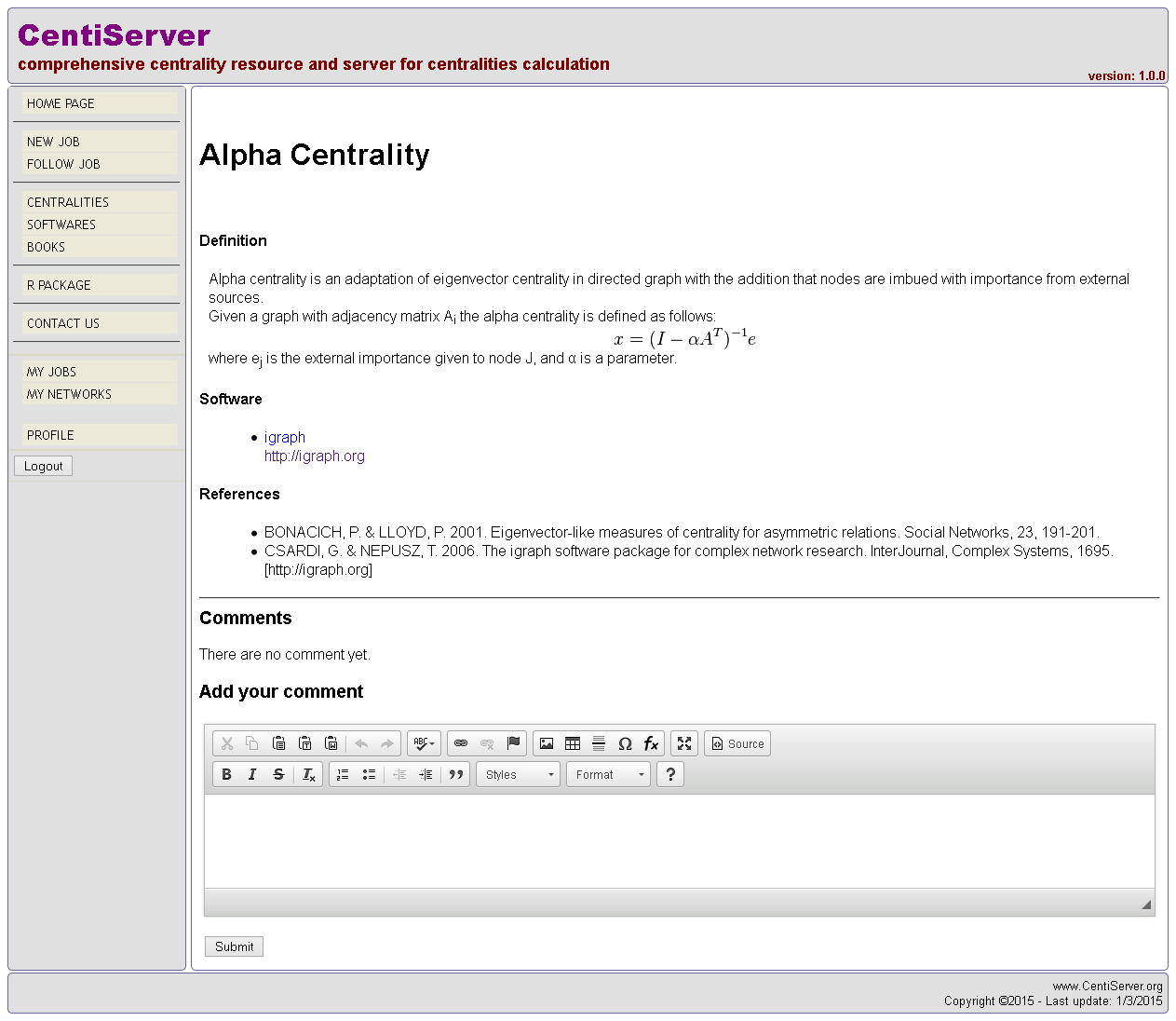


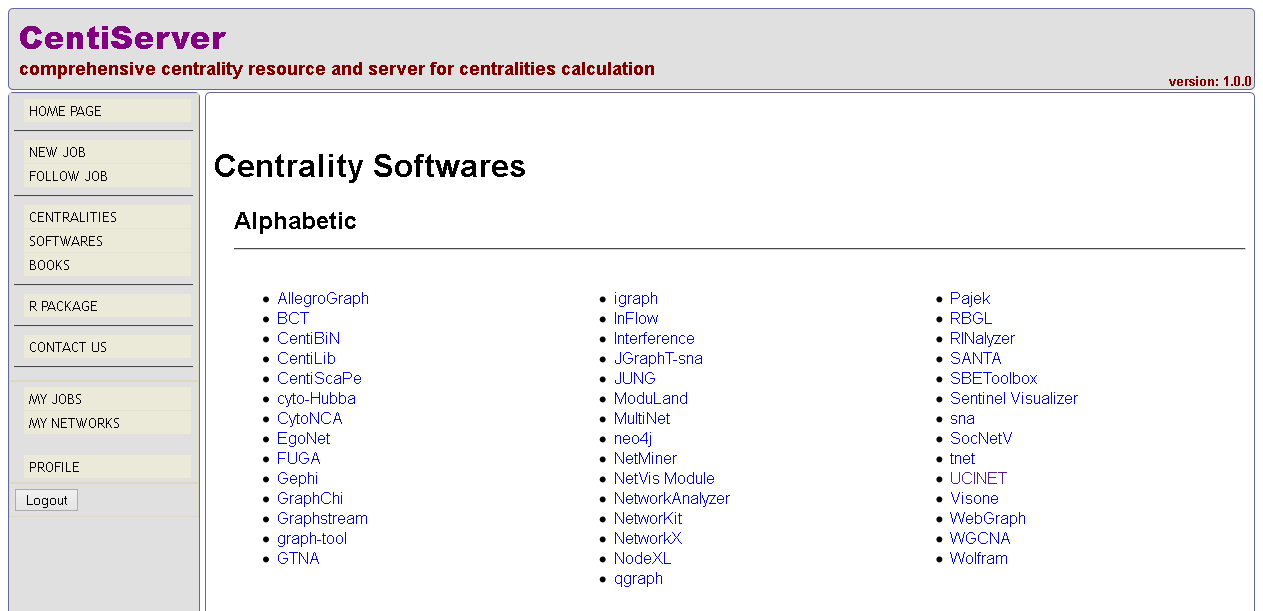


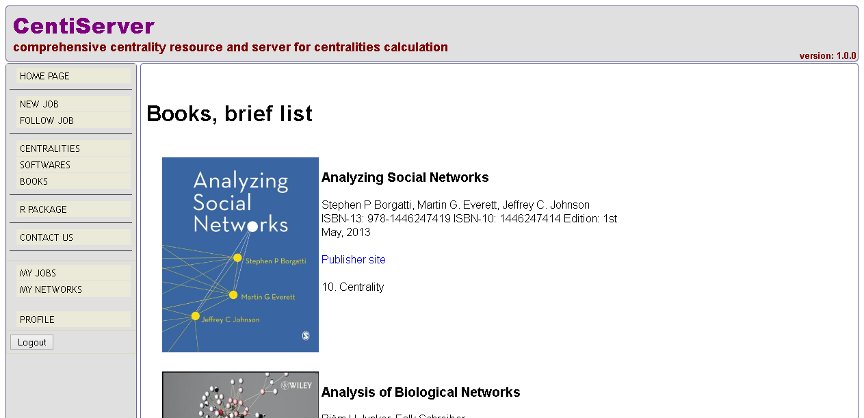


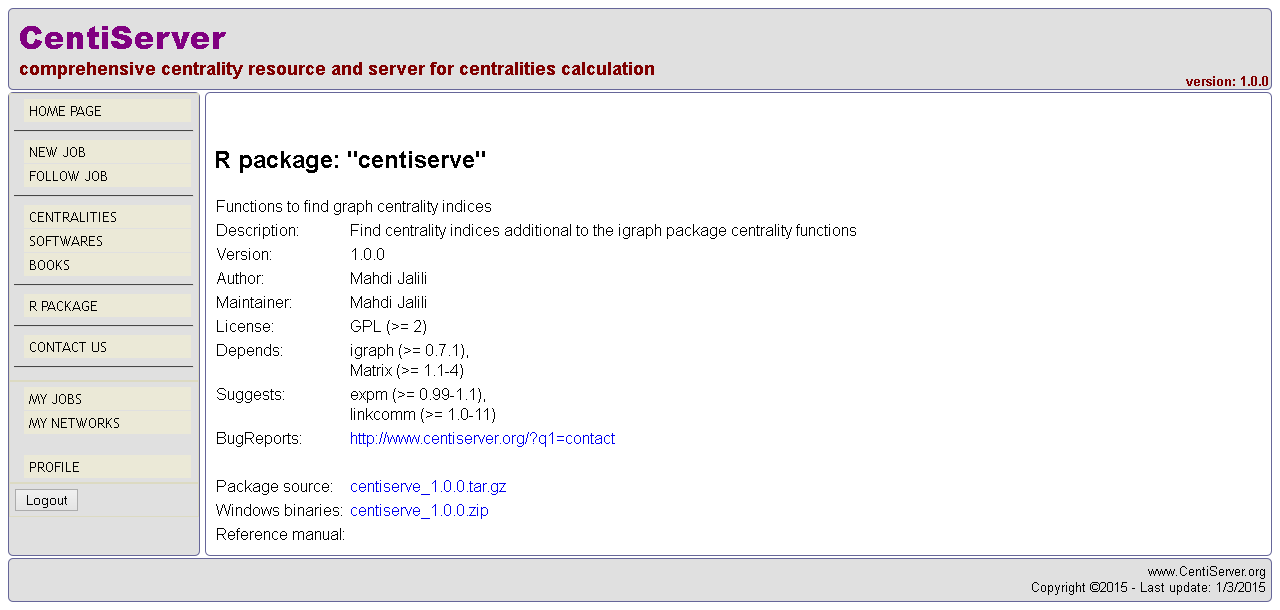


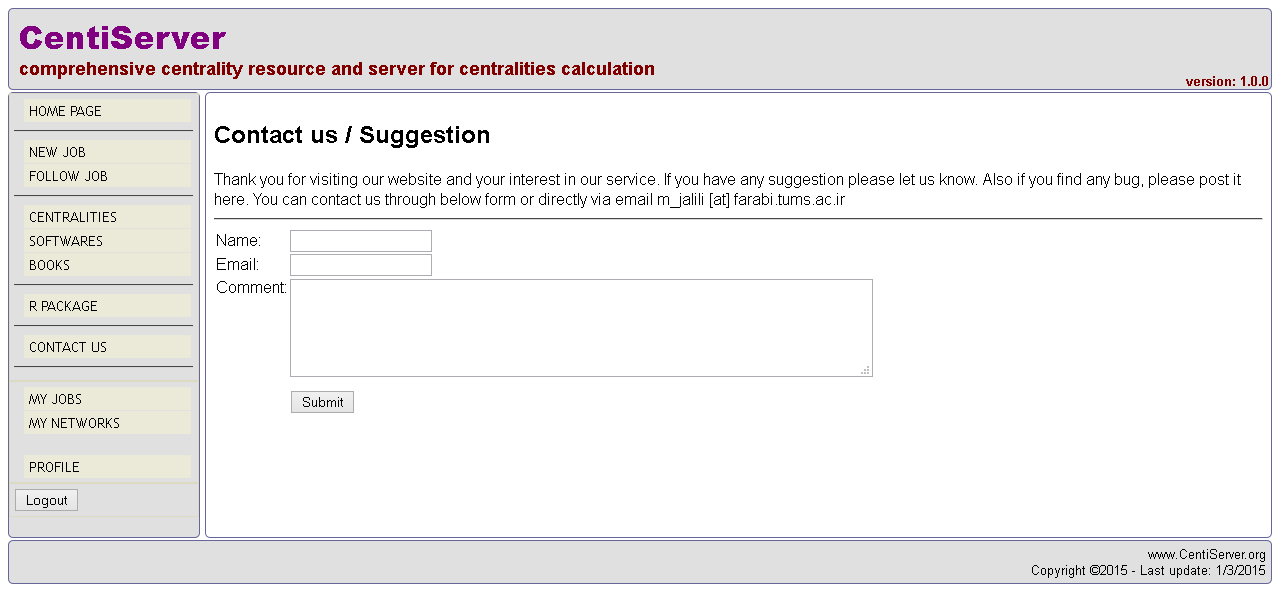


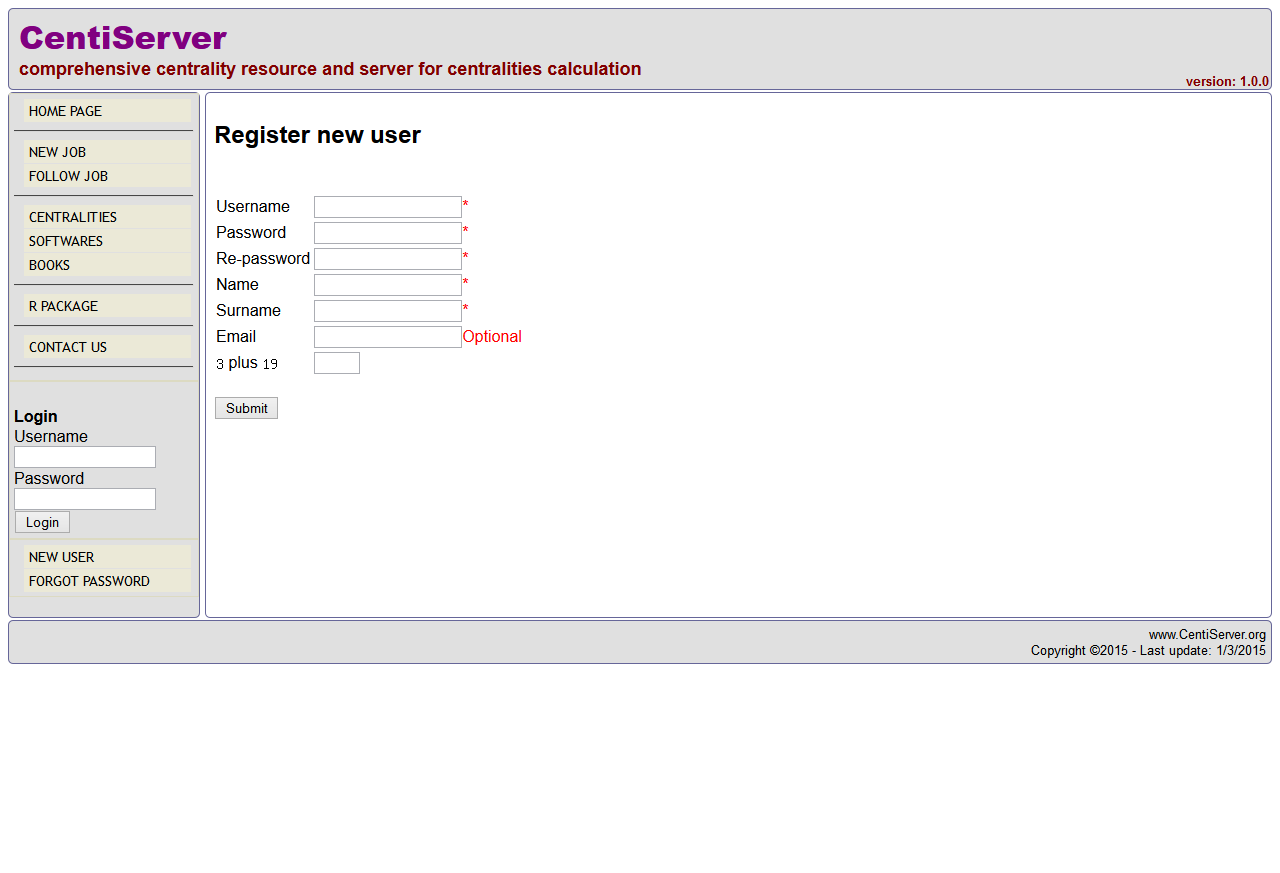

Supplement: S2 File — (DOC) [file pone.0143111.s002.doc]
